# Current landscape of personalized medicine adoption and implementation in Southeast Asia

**DOI:** 10.1186/s12920-018-0420-4

**Published:** 2018-10-26

**Authors:** Huey Yi Chong, Pascale A. Allotey, Nathorn Chaiyakunapruk

**Affiliations:** 1 School of Pharmacy, Monash University Malaysia, Subang Jaya, Selangor Malaysia; 2 United Nations University International Institute for Global Health, Kuala Lumpur, Malaysia; 3 Center of Pharmaceutical Outcomes Research (CPOR), Department of Pharmacy Practice, Faculty of Pharmaceutical Sciences, Naresuan University, Phitsanulok, Thailand; 4 School of Pharmacy, University of Wisconsin, Madison, USA; 5 Asian Centre for Evidence Synthesis in Population, Implementation and Clinical Outcomes (PICO), Global Asia in the 21st Century (GA21) Platform, Monash University Malaysia, Subang Jaya, Malaysia

**Keywords:** Personalized medicine, Pharmacogenomics, Implementation, Southeast Asia

## Abstract

**Background:**

The emergence of personalized medicine (PM) has raised some tensions in healthcare systems. PM is expensive and health budgets are constrained - efficient healthcare delivery is therefore critical. Notwithstanding the cost, many countries have started to adopt this novel technology, including resource-limited Southeast Asia (SEA) countries. This study aimed to describe the status of PM adoption in SEA, highlight the challenges and to propose strategies for future development.

**Methods:**

The study included scoping review and key stakeholder interviews in four focus countries – Indonesia, Malaysia, Singapore, and Thailand. The current landscape of PM adoption was evaluated based on an assessment framework of six key themes – healthcare system, governance, access, awareness, implementation, and data. Six PM programs were evaluated for their financing and implementation mechanisms.

**Results:**

The findings revealed SEA has progressed in adopting PM especially Singapore and Thailand. A regional pharmacogenomics research network has been established. However, PM policies and programs vary significantly. As most PM programs are champion-driven and the available funding is limited, the current PM distribution has the potential to widen existing health disparities. Low PM awareness in the society and the absence of political support with financial investment are fundamental barriers. There is a clear need to broaden opportunities for critical discourse about PM especially for policymakers. Multi-stakeholder, multi-country strategies need to be prioritized in order to leverage resources and expertise.

**Conclusions:**

Adopting PM remains in its infancy in SEA. To achieve an effective PM adoption, it is imperative to balance equity issues across diverse populations while improving efficiency in healthcare.

**Electronic supplementary material:**

The online version of this article (10.1186/s12920-018-0420-4) contains supplementary material, which is available to authorized users.

## Background

Driven by a spectrum of genomic breakthroughs, personalized medicine (PM) is the central theme across healthcare systems, mainly in the United Kingdom (UK), the United States (US), and the European Union (EU). Commonly, it is known as an emerging field in which the application of specific biological markers, often genetic [1, 2], enables diagnosis and disease management to be more accurately targeted at the individual patient [3]. By providing “the right treatment to the right patient at the right time”, a paradigm shift in healthcare from the current “one-size-fits-all” to a more personalized approach can be realized.

Despite its high cost, many countries have started to adopt this novel PM technology including Southeast Asia (SEA). At the same time, these healthcare systems are striving towards universal health coverage (UHC) to ensure everyone has access to needed health services, without undue financial hardship, financial constraint remains as one of the main challenges in attaining and maintaining UHC [4, 5]. Adopting expensive PM would place increasing strain on the already constrained healthcare budget, further raises concerns on equitable, affordable and sustainable of such healthcare delivery to populations in the context of UHC. Thus, the need of an efficient healthcare delivery is highly critical.

Based on valuable experience from “early adopters”, several pressing issues were highlighted: limited availability of evidence for clinical utility; lack of awareness among providers, patients and families; limited access to genetic testing; lack of reimbursement for genetic testing; the need for real-time, point-of-care integration of test results with the electronic health record (EHR) and clinical decision support (CDS) tools; and ethical, legal, and social concerns [6–9]. Primarily, both economic challenges and operational issues present the most significant obstacles to the development of PM adoption and implementation [10, 11]. To harness PM in SEA, changes at multilevel in the healthcare systems are essential to improve the quality of patient care and health system productivity.

Given the complexities of PM adoption and implementation, this study aimed to describe the current policy and program approaches by different stakeholders in SEA to promote PM adoption and implementation, highlight the challenges and to propose strategies for future development.

## Methods

The overall methodology of the study was adapted from Shafie et al*.* [12] and Lim et al [13]. The human research ethics approval was granted by Monash University Human Research Ethics Committee (reference number: 2017–8622).

### Study settings

Southeast Asia, a region of 11 countries with 2.2 billion people [13]. Based on the World Bank income categories, this region consists of high-, upper middle-, lower middle-income countries [14]. A total of 4 focus countries were selected and reviewed in this study – Indonesia, Malaysia, Singapore, and Thailand. This was mainly due to the capacity for regional cooperation and openness, and fairly developed national health programs in these countries.

### Assessment framework

In this study, the current landscape of PM adoption and implementation in SEA was evaluated using an assessment framework. This framework focused on six major key themes – (i) healthcare system, (ii) governance, (iii) access, (iv) awareness, (v) implementation, and (vi) data (Additional file 1). These were identified through multiple sources from reference countries (the UK, the US, the EU, Australia, and Canada). Sources included PM initiative/strategic plan, published government report, and published literature. The framework was further supplemented using the World Health Organization (WHO) framework for action in strengthening health systems [15] and the WHO action framework for the Western Pacific region towards UHC [4].

Personalized medicine is not a rigid concept, but rather encompasses different types of technology. Given the diversity and heterogeneity of PM programs available in SEA, six PM programs – targeted oncology therapy/companion diagnostic, pharmacogenomics (PGx) testing, newborn screening, prenatal diagnosis, cancer risk screening, and advanced multigene sequencing were selected to further evaluate the current financing and integration mechanisms in SEA’s healthcare system.

### Data sources

A scoping review was undertaken using electronic databases – PubMed, EMBASE, CINAHL, Cochrane Library, and Web of Science, as well as appropriate grey literature, e.g. Ministry of Health websites, published policy documents, and national guidelines, as well as Google Scholar. The search strategy used is presented in Additional file 2. Considering that the completion of human genome sequencing in April 2003 [16], searches were restricted by date from 1 January 2004 to 31 January 2017, further limited to the focus countries. Any literature that reported on the PM adoption was included. Multiple sources of evidence with no restriction on type of study design were included in this review. Data related to the key themes were extracted.

A semi-structured interview with key opinion leaders (KOLs) in four focus SEAs was conducted. These KOLs were researchers and clinicians, identified and contacted through government channels and expert connections. A snowball sampling technique was used in which other potential participants were nominated by initial through connections in the field. An interview guide was developed with open questions surrounding the key themes and any obvious gap collected from the literature review. This guide was validated to address any ambiguity or duplicate. The interviews were conducted from May to July 2017. Interviewees were briefed about the objectives of the research and gave their informed consent to participate and for the interview to be audio-recorded. Country-specific information related to the key themes was extracted from the interview transcript to supplement the findings derived from the review.

In this study, we focused on the current status of PM adoption and implementation in the public healthcare settings. Findings in SEA from literature and interview were then benchmarked with that of any reference countries.

## Results

The literature search yielded 9537 articles across 5 electronic databases. After removal of duplicates, there were 9503 unique articles. A total of 698 articles were selected for further screening and 30 articles were included in this study. Furthermore, a number of relevant articles, government guidelines/reports and websites were found from other sources and included in the review - eight journal articles, six government guidelines/reports, and three websites. The list of included articles is presented in Additional file 3. As for the KOL interviews, 11 KOLs from four focus countries participated in the interview. They were researchers (*n* = 6, 55%) and clinicians (*n* = 5, 45%) involved in the provision of PM service in their country. At least 2 KOLs were interviewed from each country. The sociodemographic characteristics of the KOLs interviewed are presented in Table 1.

**Table 1:** Sociodemographic characteristics of key opinion leaders participated (*n* = 11) in the interview

Sociodemographic variables	N	%
Gender (*n* = 11)		
Male	6	55
Female	5	45
Country (*n* = 11)		
Indonesia	2	18
Malaysia	4	36
Singapore	2	18
Thailand	3	28
Professional role (*n* = 11)		
Clinician	5	45
Researcher	6	55

In general, the four focus SEA countries have made progress towards PM adoption. However, significant heterogeneity of the PM adoption and implementation in terms of the key themes was noted (Table 2). Among them, Singapore and Thailand have made remarkable efforts in PM adoption – an upcoming national PM initiative in Singapore aiming for a nationwide PM adoption, while Thailand has progressed ahead with the introduction of PGx card for patients. On the other hand, PM-related activities remain largely in the research stage and/or as special clinical services in selected national hospitals and university hospitals in Malaysia and Indonesia. A regional research group – South East Asian Pharmacogenomics Research Network (SEAPharm) has been established with six Asian countries (Japan, Korea, Indonesia, Malaysia, Taiwan, and Thailand), and the latest additions of Singapore and Vietnam. This was initiated by Thailand and Japan – Thailand Centre of Excellence for Life Sciences (TCELS), Department of Medical Sciences in Ministry of Public Health (MOPH), Ramathibodi Hospital in Mahidol University, and Riken Centre for Integrative Medical Sciences.

**Table 2:** Current PM adoption in four focus SEA countries based on six key themes

Key themes	Indicators	Indonesia	Malaysia	Singapore	Thailand
Healthcare system	General				
	GDP per capita (Current USD)	3500	11,306	56,007	5970
	Healthcare financing system	Social health insurance	Tax-funded	Mixture from tax revenue, insurer and patient	Social health insurance
	Healthcare expenditure per capita (USD)	99	456	2752	360
	THE (% of GDP, 2014)	2.8	4.2	4.9	6.5
	Health coverage (%)	48	100	100	98
	OOP health expenditure (% of THE, 2014)	46.9	35.3	54.8	7.9
	PM-specific				
	Presence of PM-related healthcare service delivery				
	1. Targeted oncology therapy	Yes	Yes	Yes	Yes
	2. PGx testing	No^a^	No^a^	Yes^b^	Yes^c^
	3. Newborn screening	Yes (congenital hypothyroidism)	Yes (congenital hypothyroidism, G6PD)	Yes (congenital hypothyroidism, G6PD, inherited metabolic disorders)	Yes (congenital hypothyroidism, thalassemia, phenylketonuria)
	4. Cancer risk screening	No^a^	Yes^d^	Yes^d^	No^a^
	5. Advance genome sequencing	No	No	Yes^e^	Yes^e^
	Presence of PM-related healthcare workforce				
	1. Medical geneticist	Yes (NR)	Yes (9)	Yes (6 + 2 cancer geneticist)	Yes (11)
	2. Genetic counsellors	Yes (NR)	Yes (2)	Yes (≈10)	No
	Financing mechanism				
	1. Capacity-building	NR	NR	NR	NR
	2. Infrastructure	NR	NR	NR	NR
	3. Research	Cyclic grants from government, university, international collaborators	Cyclic, one-off grant from government, university	Funding from A*STAR	Cyclic, one-off grant from government, university
Governance	National strategy/plan	No	No	National PM initiative (in progress)	No
	Comprehensive PM legislation/guideline	No	No^f^	No^f^	No
	Ethical, social, legal framework on PM provision	No	No	PM-specific provision standard is in progress	No
	Ethical, social, legal framework for genetic data	No	Yes, but no laws related to genetic discrimination by insurance companies	PM-specific standard is in progress^g^	Yes, but no laws related to genetic discrimination by insurance companies
	National PM research centre or large-scale research initiative	No	No	GIS; POLARIS; SAPhIRE; PRISM	PGx projects by TCELS
	Direct to consumer test legislation or code of conduct	No	No	Bioethics Advisory Committee recommendations	Existing consumer law
	PM working group with multiple stakeholders	No	No	Yes	Yes
Access	HTA body	Yes Major hospitals	Yes National	Yes National	Yes National
	PM-specific HTA framework	No	No	No^h^	No
	Multi-stakeholder decision-making group	Yes	Yes	Yes	Yes
Awareness	Patient support/advocacy groups	No	Yes	Yes, but not specific	Yes, but not specific
	Efforts to increase public awareness	Yes	Yes	Yes	Yes
	Patient involvement in healthcare and/or research	Low in research	Low in research	Moderate in research	High in research
Implementation	Centre of excellence/leading institute in PM service	Dr Cipto Mangunkusumo Hospital; Center for Biomedical Research in Diponegoro University	Institute of Medical Research	PRISM	Ramathibodi Hospital; Khon Kaen University Hospital; Siriraj Hospital
	Education and training for PM and non-PM specialized healthcare workforce including medical school	Yes	Yes	Yes	Yes
Data	EHR	No	No	Yes	Yes
	Biobank	Hospital-level biobanks	Malaysian Cohort Biobank; UKMMC-UMBI Biobank	SingHealth Tissue Repository; National University Health System Tissue Repository	Hospital-level biobanks
	Database	Indonesian National Genetic Database	Malaysian Human Variome Project	Singapore Genome Variation Project database; Singapore Human Mutation/ Polymorphism Database; Singapore PGx Portal	Thailand Mutation and Variation database
	Patient registry with genetic/genomic data	No	Thalassemia Registry	Singapore Polyposis Registry; Thalassemia Registry; National Birth Defect Registry	No

Notes

^a^ Available through special request

^b^ HLA-B*15:02 screening is mandatory in Singapore. UGT1A*6 and UGT1A1*28 testing are available in National Cancer Centre

^c^ HLA-B*15:02 screening is routinely practised in major hospitals. A variety of other PGx testings are available as service in several university hospitals

^d^ *BRCA* screening is available in a few national/university/ specialised hospitals: University Malaya Medical Centre, Hospital Kuala Lumpur in Malaysia; National Cancer Centre Singapore, National University Hospital in Singapore

^e^ Next-generation sequencing is available in leading hospital: SingHealth-POLARIS in Singapore; Ramathibodi Hospital in Thailand

^f^ Some degree of ethical oversights are governed under the existing national medical genetics service and/or genetic testing guideline

^g^ Includes informed consent, security and confidentiality of information, and disclosure of test results to third parties outside direct healthcare providers

^h^ Genetic test is evaluated as medical device

Abbreviations

A*STAR Agency for Science and Technology Research, *EHR* electronic health record, *GDP* gross domestic product, *GIS* Genome Institute of Singapore, *HTA* health technology assessment, *NR* not reported or insufficient information, *OOP* out-of-pocket, *PGx* pharmacogenomics, *PM* personalized medicine, *POLARIS* Personalized OMIC Lattice for Advanced Research and Improving Stratification, *PRISM* SingHealth Duke-NUS Institute of Precision Medicine, *SAPhIRE* Surveillance and Pharmacogenomics Initiative for Adverse Drug Reactions, *TCELS* Thailand Centre of Excellence Life Sciences, *THE* total health expenditure, *UKMMC* University Kebangsaan Malaysia Medical Centre, *UMBI* UKM Medical Biology Institute, *USD* United States dollar

### Healthcare systems

The healthcare delivery capabilities and financing among these focus countries vary. Majority of the countries have relatively high healthcare coverage, 98–100%. However, an alarmingly high out-of-pocket (OOP) expenditure of more than 40% was noted in Indonesia (46.9%) and Singapore (54.8%). To date, a national PM initiative is yet to be implemented except Singapore, a high-income country (HIC) is in the drafting process. Furthermore, Thailand, a developing low- and middle-income country (LMIC), has been engaging in PGx research since 2004 through the Thai PGx Project with investments from TCELS.

Besides targeted oncology therapy/companion diagnostics and newborn screening are delivered as routine practice in four focus countries, the availability of other PM programs is limited, either in a few major tertiary/university hospitals or in the research stage. Interestingly, PGx testing of HLA-B*15:02 prior to carbamazepine (CBZ) initiation has been incorporated as the standard of care in Singapore and Thailand.

In terms of PM-specific healthcare providers, the workforce remains insufficient – 2 cancer clinical geneticist in Singapore, 6 (Singapore) to 22 (Thailand) clinical/medical geneticists, and approximately 2–20 genetic counsellors in Malaysia and Singapore. At present, genetic counsellor is not commonly available as there is no official position within Malaysia’s Ministry of Health (MOH), while no formal training of genetic counsellor in Thailand as well.

### Governance

The absence of governance and legislation in the provision of PM and genetic data, neither specific guideline nor regulation is formalized among all focus countries. Nevertheless, there are a few frameworks related to genomics/genetics already in place. As early as 2004, Thailand promulgated the National Biotechnology Policy Framework 2004–2009, where genomics and bioinformatics were identified as key strategy areas for improving the health of the Thai people, developing new bio-businesses as well as creating a self-sufficient economy. In Malaysia and Singapore, a national guideline on medical genetics service and/or genetic testing provides some degree of ethical oversights on the use of genetic in healthcare.

Since genetic data is categorized as medical data, thus its confidentiality is governed under the existing legislation in each country. In addition, recommendations on issues – the protection of privacy and confidentiality of genetic information in biomedical research, and access to genetic information by employers and insurers are governed under the national guideline aforementioned in Malaysia and Singapore. Only Singapore is drafting a PM-specific provision standard that incorporates the ethical, social, legal framework of this provision in healthcare and genetic data.

Local PM research plays an integral role in improving the ability to respond to local disease threats by translating locally discovered genomic biomarkers into clinical applications. In all focus countries, research is mostly conducted at the institutional level, with the support of cyclic grants from government, university, or international collaborators. Furthermore, insufficient funding remains one of the main challenges to sustain PM research. Only a few large-scale research initiatives in Singapore and Thailand have been funded with considerable governmental financial supports such as Singapore’s Agency for Science and Technology Research (A*STAR) and Thailand’s TCELS, a public organization under the Thai Ministry of Science and Technology.

In Singapore, there are several major players – government agency, clinician, and researcher/academia driving the development of PM adoption. Since 2000, a cascade of major development strategies ranging from research to clinical application marks an important milestone in the roadmap of Singapore’s PM adoption (Table 3). Notably, the SingHealth Duke-National University Singapore Institute of Precision Medicine (PRISM) is one of key drivers underpinning the upcoming national PM initiative. Under PRISM’s initiative, a multi-dimensional database, “SPECTRA” will be developed from 5000 healthy Asian volunteers that incorporates genomic, clinical, lifestyle, and imaging data, subsequently to serve as reference database for disease-oriented studies by specialty centers and across the nation.

**Table 3:** Major milestones in the development of PM adoption in Singapore

Year	Organisation/ Initiative	Funder/Collaboration	Aim
2000	GIS^i^	A*STAR	To use genomic sciences to achieve improvements in human health and public prosperity
2013	POLARIS	GIS, SingHealth	To deliver better patient outcomes through research within SingHealth institutions^j^
2014	SAPhIRE	BMRC and the HSA, GIS, and the Translational Laboratory for Genetic Medicine	To develop an active surveillance network for ADR monitoring and discovery of genomic biomarkers that are predictive of specific ADRs
2015	PRISM	SingHealth and Duke-NUS Medical School	To drive, promote and standardize the use of PM and Precision Health for improving patient care, focusing on diseases relevant to Asian populations

Notes

^i^ National flagship program for the genomic sciences

^j^ This includes Singapore General Hospital, National Cancer Centre Singapore, Singapore National Eye Centre, and NUS Health System

Abbreviations

A*STAR Agency for Science and Technology Research, *ADR* adverse drug reaction, *BMRC* Biomedical Research Council, *GIS* Genome Institute of Singapore, *HSA* Health Sciences Authority, *NUS* National University of Singapore, *POLARIS* Personalised OMIC Lattice for Advanced Research and Improving Stratification, *PRISM* SingHealth Duke-National University Singapore Institute of Precision Medicine, *SAPhIRE* Surveillance and Pharmacogenomics Initiative for Adverse Drug Reactions

While in Thailand, a genotyping initiative was launched in consistence with the Thai biotechnology policy framework. Catalyzed by an initial investment of THB 120 million by TCELS, six PGx projects were initiated on diseases common among Thai – human immunodeficiency virus, CBZ- and allopurinol-induced severe cutaneous adverse reactions (SCARs), acute childhood lymphoblastic leukemia, oncology chemotherapy, aspirin responsiveness, and thalassemia-hemoglobin E.

As part of the SEAPharm’s collaborative effort, “100 Pharmacogene Project” has been recently announced. This will involve genomes sequencing from 1000 patients from 10 SEA participating members. A pharmacogene database will be produced consisting of genetic variations of 100 pharmacogenes associated with drug efficacy and safety among SEA populations. At the same time, a research guideline will be proposed to support this regional, large-scale research work.

In the UK, PM adoption in the National Health Service (NHS) has been long supported by high-level political endorsement [17]. A national strategic vision has been set out by the Human Genomics Strategy Group through specific recommendations on steps needed for the NHS to benefit from PM adoption. These recommendations include (i) translating genomic innovation into quality-assured care pathways; (ii) developing an equitable, affordable, and high-quality service delivery; (iii) setting up the bioinformatics platform; (iv) preparing the workforce; (v) developing the legal and ethical framework; and (vi) engaging the public and building awareness [18]. Furthermore, specific funding has been allocated for development of policies on genomics in healthcare, approximately €200,000 per year in the UK [19]. At present, PM research continues to receive substantial investment from the federal government in the UK, the US, and Canada [18, 20, 21]. Similarly, in Australia, the recognition of strong leadership and governance in PM adoption will be substantiated, in consistence with the call in the upcoming National Health Genomics Policy Framework as the most critical priority area to be addressed. Consequently, improvements in all other priority areas of the Framework would be achieved accordingly. With the joint national leadership and strong governance arrangements at the state, national, international levels, nationally unified directions will be provided to promote consistent and coordinated implementation of activities across Australia in the integration of genomics into the Australian health system [22].

### Access

Despite the upsurge of health technology assessments (HTAs) and the corresponding bodies in four focus countries at the national or hospital level, current approaches to economic evaluation to support decision making are largely focused on reimbursement of drug. Given the absence of specific HTA framework for PM interventions, the current HTA state to comprehensively guide decisions on the reimbursement and coverage of PM is limited. Among the four focus countries, only Singapore’s Agency of Care Effectiveness is currently evaluating genetic testing as medical device.

In addition, many jurisdictions have adopted a cost-effective threshold to guide the decision-making process [23]. It is revealed that the application of a general threshold of THB 160,000 per quality-adjusted life year gained for oncology PM in Thailand [24].

The Pharmaceutical Benefits Advisory Committee in Australia has introduced a guideline for HTA submission of hybrid technologies and co-dependent technologies. This includes clinical evaluation on evidence related to prognostic effect of the biomarker, performance and accuracy of the proposed test, change in clinical management, and clinical evaluation of the co-dependent technologies, as well as economic evaluation that captures both accurate and inaccurate test results among eligible population [25, 26]. While the UK Genetic Testing Network Steering Group has endorsed and adapted the analytic validity, clinical validity, clinical utility, and ethical, legal and social issues as core principles to produce a “Gene Dossier” for evaluating genetic tests in the NHS [27]. Furthermore, the UK National Institute for Health and Care Excellence evaluates companion diagnostics using either the Diagnostics Assessment Program or the Technology Appraisal process with the focus of incorporating clinical evidence in terms of the test’s predictive value and ability to identify the eligible patient population [28, 29].

### Awareness

A lack of specific information on genetic/genomic available to the public and patients, corresponding to the poor awareness of PM, except for oncology-related information from various sources – digital media, the existing patient advocacy and support groups such as information dissemination on *BRCA1* and *BRCA2* genetic screening by the Breast Cancer Singapore, awareness campaigns on cancer and related research by Cancer Research Malaysia including *BRCA* genetic screening, as well as the Hereditary Breast and Ovarian Cancer Campaign by Cancer Advocacy Society of Malaysia. Through online education, information related to targeted oncology therapy for the treatment of breast cancer and *HER2* in breast cancer is made available by the National University Hospital Singapore and the “Yayasan Kanser Payudara Indonesia” (Indonesia Breast Cancer Association). Beyond oncology, there is a general trend that education efforts are uncommon and clinicians are often the main source of information at the point-of-care.

In Thailand, education programs on PGx testings have been actively carried out by the TCELS and Ramathibodi Hospital. As a result, several PGx books and reports were released for decision makers, clinicians, scientists, and layman. Notably, an educational PGx mobile application is developed to raise the awareness on PGx among patients and public.

As part of the EU’s International Consortium for PM (ICPerMed) Action Plan, a European Knowledge Platform will be developed to improve both health and digital literacy using web-based and social media instruments over short and medium term. Best practices of patient engagement approaches will be developed to enhance patients’ experience and their need to participate and make informed decision on their healthcare, as well as their involvement in all phases of PM’s research and development [30]. Several important European organizations are aiming to promote the ideas of PM. Among them, the European Alliance for Personalized Medicine collaborates with health experts and patient advocates through developing case studies, organizing workshops, education, training, and communication [31].

### Implementation

Overall, a few major healthcare institutes have been leading in the provision of PM programs as their service in four focus countries. Unlike targeted oncology therapy/companion diagnostics with supports from manufacturers, the availability and integration of other PM programs are mainly driven by successful research projects previously conducted by local champions. This corresponds to the availability of these services are mainly in the national capital or university hospitals.

The availability of clinical expertise varies widely across the region with an overall significant lack of genetic expertise. Although genetics/genomics are integrated into the medical school curricula, continuing education and training for healthcare providers remains to be insufficient in all focus countries as there is neither formal training program nor capacity building program in place.

In Thailand, the launching of PGx card by Ramathibodi Hospital represents an important progress in PM adoption. It aims to summarize patients’ *HLA* gene variant information predicting risk of developing SCARs from specific drugs.

In the UK, NHS England specialized services has progressed in setting up a network consisting of genomic technology centers, biomedical diagnostic hubs and regional genetics centers. This enables NHS to advance the use of genomic information into mainstream clinical medicine. New education and training programs have been developed and implemented across the UK as part of the Department of Health Modernizing Scientific Careers program. The programs aim to ensure training for whole NHS workforce on genomics and PM [18].

### Data

Genetic/genomic data is complex and its significance to the individual patient changes throughout the patient’s life. The EHR implementation is an important tool in PM adoption especially the support using a CDS enables clinicians in making informed decisions at the point-of-care. However, a nationwide EHR system is available in Singapore and Thailand.

Furthermore, patient registry is critical in the management of genetic diseases. Although there are patient registries with genetic data in Malaysia and Singapore, only Singapore Polyposis Registry and Singapore Thalassemia Registry are utilized to facilitate identification, surveillance and management of families and individuals at high risk of colorectal cancer and thalassemia. For research purposes, several genetic databases and/or biobanks have been established, either at the national or hospital level in all focus countries.

In the UK, work is underway in consistence with the NHS Paperless 2020 program through NHS digital. This aims to create a fully interoperable EHR (incorporating genomic, clinical and diagnostic, medicines, and lifestyle data), supported by information sharing and data linkage [32]. In the early 2000s, the Electronic Medical Records and Genomics Network, a US National Human Genome Research Institute–funded consortium that combines genomic with EHR for large scale, high-throughput genetic research in support of implementing genomic medicine [33]. It has demonstrated that data captured through routine clinical care are sufficient to generate findings for large-scale genetic research. Data harmonization, an important issue needs to be addressed when dealing with a spectrum of big data generated from PM research. As part of the EU’s ICPerMed Action Plan, research will be undertaken to ensure the appropriate alignment of existing and future datasets with important information for PM, thus promoting the development and implementation of existing as well as innovative PM strategies along the value chain [30].

### Comparison of the financing and implementation of PM programs

Among six PM programs investigated, information gathered related to 3 of these programs was sufficient to make a meaningful comparison of their financing and integration mechanisms (Table 4). Thus, this comparison included (i) trastuzumab/*HER2* testing for targeted oncology therapy, (ii) HLA-B*15:02 screening for PGx testing, and (iii) congenital hypothyroidism screening for newborn screening. Unlike trastuzumab/*HER2* testing and congenital hypothyroidism screening are provided routinely in all focus countries, HLA-B*15:02 screening is available as the standard care in Singapore and Thailand, but not in Malaysia and Indonesia despite the high allele frequency in SEA (> 15%) [34].

**Table 4:** Comparison of the financing and integration mechanisms of three most common PM programs in SEA

Indicators		Targeted oncology therapy and companion diagnostic	Pharmacogenomics testing	Newborn screening
		Trastuzumab	HLA-B*15:02	Congenital hypothyroidism
Availability as routine practice	Yes	Indonesia Malaysia Singapore Thailand	Singapore (nationwide) Thailand (major hospitals)	Indonesia (10 provinces in 2017) Malaysia Singapore Thailand
	No	–	Malaysia (special request) Indonesia (research)	–
Stakeholder that initiated the PM program	Pharmaceutical company	Indonesia Malaysia Singapore Thailand	–	–
	Clinicians	–	Singapore Thailand	Indonesia Malaysia Singapore Thailand
Financing mechanism	Covered in national health program	Indonesia (*HER2* testing as OOP expenses) Malaysia Thailand	Thailand (Cap at THB1000)	Indonesia (Limited budget) Malaysia Thailand
	Partial subsidy by the national health programs	Singapore (under Medical Assistance Fund scheme)	Singapore (Up to 75% subsidy)	Singapore (60% subsidy)
Monitoring framework	Clinical outcome	Malaysia (in future plan) Thailand	Singapore	–
Integration in healthcare	Change in local clinical practice guideline	Indonesia Malaysia Singapore Thailand	Thailand Singapore	Indonesia Malaysia Singapore Thailand
	Availability of CDS in EHR	NR	Singapore Thailand (some hospitals)	NA
Presence of healthcare information	Physician	Indonesia Malaysia Singapore Thailand	Singapore Thailand	Indonesia Malaysia Singapore Thailand
	Patient	Singapore	Thailand	Indonesia Malaysia Singapore Thailand

Abbreviations

*CDS* clinical decision support, *EHR* electronic health record, *NA* not applicable, *NR* not reported or insufficient information, *OOP* out-of-pocket, *THB* Thai Baht

Trastuzumab was introduced into the market with enormous manufacturer support including, but not limited to the sponsorship of *HER2* testing and the readily available clinical and economic evidence. The remaining two PM programs are champion-driven, predominantly by clinicians. The HLA-B*15:02 screening was initiated by the Health Sciences Authority (HSA)‘s pharmacovigilance unit in Singapore, while in Thailand by clinicians and MOPH. The congenital hypothyroidism screening was introduced since 1990s in all focus countries except Indonesia in 2008 by the association of pediatricians. Both involved a lengthy process to advocate their adoption. Apart from HTA studies, locally-conducted observational pilot studies were employed to demonstrate the value of this screening to policymakers.

Limited funding remains a major hurdle for a widespread PM service. This is most evident in Indonesia, up to today, the financial allocation has never been adequate for all newborns to be screened for congenital hypothyroidism after 9 years of its nationwide introduction in 2008. Even though the coverage of trastuzumab/*HER2* testing by national health programs, these are publicly reimbursed with the existing, tight budget allocation in Malaysia or partially subsidized under the “Medication Assistance Fund” in Singapore. However, in Indonesia, *HER2* testing is not covered under the national health insurance scheme. As for HLA-B*15:02 screening, its reimbursement policy is highly dependent on the cost of genetic testing in Thailand (<THB 1000). While in Singapore, multi-stakeholder discussions with clinicians, HSA were held to address pertinent issues to further reduce the genotyping cost and turnaround time prior to its mandatory implementation [35]. Today, it is partially subsidized up to 75%.

The integration of the PM programs into healthcare varies significantly. For trastuzumab/*HER2* testing, its integration as a package has been largely supported by the relevant manufacturers. This usually follows with consistent, timely changes in both international and local clinical practice guidelines. This has led to a more successful PM oncology application due to the adequate level of awareness and education on PM among oncologists. On the other hand, various efforts were introduced for HLA-B*15:02 screening by MOH and clinicians, including the issuance of a “Dear Healthcare Professional Letter” on HLA-B*15:02 screening prior to CBZ as the standard of care, and its recommendation in product insert in Singapore; a warning inserted on the association of HLA-B*15:02 and SCARs with CBZ use in the Thai clinical practice guideline for epilepsy. Furthermore, to better integrate HLA-B*15:02 screening into the clinical practice, CDS within the EHR system has been deployed in Singapore and Thailand. But for congenital hypothyroidism screening, the information is made available through routine educational programs or at point-of-care by physician during an antenatal care.

To date, outcomes of all PM programs have not been monitored in a standardized framework, with clinical audits for trastuzumab in the future planning. For HLA-B*15:02 screening in Singapore, the clinical outcome monitoring was based on the number of CBZ-related SCARs reported. Since the initiation of testing in 2013, no cases have been reported to the HSA’s pharmacovigilance unit.

## Discussion

In general, there is increasing interest among clinicians and researchers in PM in SEA. However, due to functional silo of each stakeholder especially clinicians, the PM output and effort are fragmented at the institutional level. At the same time, majority of healthcare systems in SEA are still facing fundamental issues in providing basic healthcare services to all citizens. As a result, the current PM adoption is slow and haphazard across SEA. To adopt PM as a national health agenda, ensuring sustainable funding and consistent political support are central for the capacity building of expertise and infrastructure at both research and clinical settings.

From the broad overview of PM initiatives across the focus countries in SEA, we have developed the following observations and recommendations:

1. Wide variations of PM adoption and implementation across the region.
  The increasing efforts in adopting and implementing various PM programs are driven by local champions. These services are predominantly located at the more affluent parts of the country. In addition, the limited funding remains a challenge to sustain PM delivery. For the example, despite the coverage of trastuzumab under the national health program in all focus countries, *HER2* testing remains as an OOP expense in Indonesia. An equitable patient access to trastuzumab is limited as *HER2* testing is more readily affordable among the rich. It is also worth noting that there is a potential risk of financial burden among trastuzumab patients in Malaysia as its provision is highly dependent on the existing limited budget allocation. Thus, the current implementation and distribution of PM programs has the potential to widen the existing health disparities, and realization of its potential can be uneven.
  The problem with inequitable patient access may be further aggravated by the low awareness on genetics/genomics and PM among patients and public. In consistence with evidence of increasing disparities in the uptake of PM in the US, these low levels of awareness will reduce patient requests for testing, in turn decreasing the testing rate among eligible minority patients [36]. This is further supported by the lack of familiarity with PM, even in the US, 73% of individuals not having heard the term [37]. Clearly, there is a need to increase health literacy on PM’s value proposition among patients, leading to an improved in the access to PM services, as well as achieving equity in PM. Moreover, with patient empowerment in their healthcare, this helps accelerate strategies to overcome other barriers in PM adoption [38].
2. The situation across SEA countries offers substantial improvement opportunities for all aspects.
  Despite some countries have more policies and support structures in place than others, significant gaps are present for all aspects, particularly the absence of governance in PM. Effective governance at the system level is the cornerstone in shaping a robust PM policy framework. Essentially, high-level commitment from a wide range of stakeholders is critical in this interactive, multi-centric process. Thus, key stakeholders are coordinated and jointly working towards an agreed institutional goal and expectation in PM adoption.
  Another notable bottleneck to PM adoption is the lack of information and communication technology platform in this region, including the need for improved CDS capabilities in an EHR. As genomic information is complex in nature, the primary role of CDS should focus on collect, disseminate, process complex health information at the point-of-care. However, this complexity increases with the latest update from additional guidelines and the discovery of clinically important gene-drug relationships [39]. Clearly, EHR represents an essential component to be integrated, and a significant need to develop a CDS framework capable of leveraging complex information on a widespread scale.
  Furthermore, the genetics/genomics competency among the healthcare workforce is a fundamental building block in this PM transformation. Due to the current shortage of PM specialist, it is particularly important for non-PM specialized health professionals such as primary care providers to provide PM services [40]. Since 2007, the US National Coalition for Health Professional Education in Genetics has outlined a minimum set of core competencies – knowledge, skills, and attitude for all health professionals from all disciplines [41]. In the primary care settings, essential skills among non-PM health professionals include taking family histories, conducting family-history assessments, interpret results of genetic tests, provide genetics/genomics education to patients, and make appropriate referrals for genetic evaluation [41, 42]. Despite the availability of many education programs for non-PM health professionals [40], recent surveys reveal they remain unprepared at large [43–46], demonstrating knowledge gaps [47]. To address this, strengthening the existing educational/training programs is at prime time [40] to more robust, rigorous pre- and post-graduate programs integrated with genomics curriculum. It is worth highlighting that developing additional educational programs for other disciplines of nongenetic health professionals is desirable. For example, the availability of an in-house pharmacists increases the utilization of PGx testing in a primary care setting [48]. The potential leading role of pharmacist in PGx adoption is further emphasized by a KOL in our interview. Therefore, by leveraging the strengths of each healthcare discipline through a competent multidisciplinary clinical team, patient care can be improved significantly with PM advancement.
  The access of PM requires immediate attention. Several related aspects represent major challenges for PM adoption in SEA – the lack of cost-effectiveness evidence for PM, the inadequacy of HTA approach for PM, and the use of a fixed cost-effectiveness threshold. Apart from Thailand, the HTA bodies in other SEA countries are in an early stage. With substantial limited human capacity, this impedes the synthesis of local cost-effectiveness data. Nevertheless, such data is especially critical to make a nuanced decision involving the uptake of high-cost PM in resource-limited SEA countries. Moreover, the current approach used to evaluate PM have been criticized as it falls short in demonstrating the multidimensional value of PM. Due to the high cost of PM and the emerging evidence base derived from a small subgroup of patients, imprecise, unfavorable cost-effectiveness findings of PM are often resulted against a fixed cost-effective threshold [49]. As a result, the delay in patient access to PM is more pronounced with further deterioration of health outcomes in SEA. To better allocate scarce resources and allow timely patient access, several strategies are essential to improve the decision-making framework – (i) investment on capacity building on HTA [50]; (ii) refinement of the current assessment approach and tool to address the greater complexity exerts by PM [51]; and (iii) the use of multicriteria decision analysis may assist in guiding the proper valuation of PM in a consistency and transparent manner [49, 52].
3. PM adoption poses tremendous challenge across the region, as the upfront costs remain high and resources are limited.
  At present, most of the SEA region are not only prioritizing chronic and communicable diseases [12], but also facing financing challenges due to the rapid demographic transition [53]. This current situation renders a strong skepticism around costly PM adoption. An augmented concern in which the financial sustainability of current basic UHC might be undermined due to PM. Despite the plummeting cost of genome sequencing with time, the high start-up costs arising from setting up infrastructure, human development, and education and research are seen as significant obstacles [54]. Although financial stability appears to be a prerequisite for PM adoption, other LMICs such as India, Mexico, and Brazil have made remarkable strides in utilizing genomic technologies [54–57].
  In common, the foremost effort in PM adoption in these LMICs is to build research capacity. This is because the research findings from HICs may be difficult to be extrapolated due to the differences in genomic/genetic profile. In SEA, multidisciplinary collaborative works between institutions should be encouraged to avoid competition and wasteful duplicates. In addition, to facilitate the sustainable future improvement in PM research, several potential solutions are suggested. These include increasing research funding, establishing centers of excellence, encouraging international collaborations, and organizing specialized training programs [54].
4. Value recognition of PM among policymakers across SEA to drive a PM era.
  Engaging policymakers is the most salient step in this PM transformation. Political will and institutional leadership are highlighted as the key factors in developing a vision and a plan, and in the implementation of population-based genotyping initiatives in “early adopters” from HICs and LMICs [55]. With consistent and vocal high-level political support, it helps in initiating, driving, and maintaining a successful PM adoption and implementation [58].
  The interest among policymakers to adopt PM across SEA remains low due to the lack of value recognition. This can be explained by the inadequate, yet emerging evidence base of PM to convince policymakers and clinicians to change policies and practices. To reduce uncertainty among stakeholder, it begins with evidence base generation to demonstrate that PM use can improve population health outcomes in a safe, effective, and cost-effective manner. As demonstrated the extensive process of acquiring and processing new local clinical and economic evidence of three aforementioned PM programs has led to their adoption decision. Potentially, an early dialogue between policymakers and manufacturers to discuss the evidentiary requirements necessary for coverage, as well as collaborations to share research data to resolve uncertainty in genomics can be a solution [38].
  Apart from policymakers, public and patients are chief actors in increasing the momentum of PM adoption. It is worth noting that enormous pressure from general public on the government to provide safer and more effective treatments has accelerated PM adoption in the US [59, 60]. Similarly, in SEA, strengthening education efforts to public and the role of patient advocacy groups is warranted in driving the PM agenda forward.
5. There are useful lessons to be learned from global experiences to enable a more effective PM adoption and implementation into the healthcare system.
  Notwithstanding various attempts supported by high-level political endorsement, PM has yet to move into the mainstream healthcare practice, even in “early adopters”. Owing to the staggering challenges to facilitate a PM adoption [17, 61], its development as a national health agenda has been dynamic and iterative. Based on global experiences, reforms at multiple levels of the healthcare system are clearly required. Evidence shows that addressing eight interdependent components – resources, governance, clinical practice, education, testing, knowledge translation, CDS and maintenance has generated positive outcomes in the implementation of a PM service [58]. SEA countries can adopt and adapt the strategies from reference countries to roll out PM adoption, while ensuring the social, ethical, equity and economic implications are fully recognized and addressed. Most importantly, PM awareness barrier needs to be lifted at all levels of society, especially patients, healthcare providers, and policymakers.
6. There is potential to organize a multi-stakeholder and multi-country approach from research to clinical implementation of PM.
  Translating discovery into routine use in healthcare is complex. As PM is an emerging concept, varying awareness and views are reported among a plethora of key players and stakeholders [62, 63]. It is suggested that partnerships between scientific research bodies, regulatory agencies, politics, clinicians, pharmaceutical and diagnostic companies, and patients/public will be the key success factor [64]. No single stakeholder can set all the essential requirements to further drive the PM adoption and implementation in healthcare. In HICs, a multi-stakeholder working group or public-private partnership has been engaged to level up key efforts for an effective PM adoption and implementation [65–67].
  Likewise, collaborations among SEA countries are highly recommended to leverage the regional expertise and resources. Notably, SEAPharm has made several efforts to foster collaborative research in SEA especially the upcoming large-scale population genotyping presents an exciting opportunity to catalyze PM development in this region. Useful next steps include bringing together health research funders, policy-making organizations, and patient advocacy groups to coordinate the research and health policy in SEA, akin to the EU’s ICPerMed.

The study is subject to a few important limitations. First, the literature search may not have identified all articles related to PM adoption and implementation, as some of these are likely to be in the local language. Given the diversity of fragmented PM-related efforts from different stakeholders, the sample of KOLs in this study may be insufficient to capture all relevant information. Furthermore, the overall methodology undertaken in this study including the snowballing approach employed may introduce bias in the information gathered from literature and KOLs. Nevertheless, we believe an overview of major developments related to PM adoption and implementation across SEA has been presented. Despite our attempts to recruit policymakers, none participated in our interview. However, majority of KOLs participated were clinicians and researchers with some influence in the national health policy making. This is likely because we are at the early stage of PM development, thus the lack of success stories to stimulate interest and attention on PM among policymakers in SEA. Despite four SEA countries were studied, the findings especially from Indonesia could be extrapolated to other lower middle-income countries in SEA where sparse progress in PM adoption and majority of the activities are in the research stage can be expected. Although key findings and recommendations between our study with Shafie et al*.* [12] and Lim et al [13] are subtle, we undertook a broader scope of PM adoption in our study.

## Conclusions

Notwithstanding the increasing interest, we are still only at the beginning of PM adoption not only in SEA, but also worldwide. Harnessing the full potential of PM to improve health is likely a decades-long endeavor. To achieve an effective PM adoption, it is imperative to balance equity issues across diverse populations, while improving efficiency in healthcare. A number of hurdles present on the road to an optimal evidence-based PM adoption in healthcare systems. In this study, we highlight the more salient issues in SEA to be addressed as a matter of priority.

In short term, there is a clear need for value recognition at all levels of society especially policymakers with more awareness and education programs. Starting with SEAPharm, better efficiency in the evidence generation activity can be achieved through regional collaborative research efforts and international partnerships. This enables not only better leverage of resources and expertise in this region, but also triggers political interest and support towards PM. Subsequently, critical discourse about PM among policymakers should be initiated to explore opportunities and priorities.

In medium and long term, it is important to move forward with a national PM strategic plan and legislation, with sustainable public and private funding. The national agreed transparent evidentiary requirement of PM’s efficacy, safety, cost-effectiveness, analytical validity, clinical validity, and clinical utility should be introduced to support PM access with appropriate on-going research to resolve uncertainty. At the country and regional level, a multi-stakeholder working group consisting of policymakers, clinicians, researchers, and patient advocacy groups is essential to step up coordination efforts to allow synergies in the preparation of PM adoption. Among the many initiatives needed to integrate PM into person-centered healthcare, setting up centers of excellence, EHR infrastructure, as well as capacity-building among healthcare providers are critical starting points. Finally, SEA should address the ethical, legal, social, and equity concerns following PM adoption and genetic data. Periodic monitoring and evaluating the clinical, ethical, social, and equity outcomes should be included to indicate the performance of PM delivery. With themes emphasized above, the perspective to formulate robust recommendations for SEA is provided to guide future public health practice in an era of PM.

## Additional files

Additional file 1: Search strategy used in the scoping review. (DOCX 15 kb)

Additional file 2: Six key themes in the assessment framework. (DOCX 18 kb)

Additional file 3: List of articles/government documents/websites included in the scoping review. (DOCX 23 kb)

## Abbreviations

A*STAR: Agency for sciences and technology research
CBZ: Carbamazepine
CDS: Clinical decision support
EHR: Electronic health record
EU: European Union
HER2: Human epidermal growth factor receptor 2
HIC: High-income country
HLA: Human leukocyte antigen
HSA: Health sciences authority
HTA: Health technology assessment
ICPerMed: International consortium for personalized medicine
KOL: Key opinion leader
LMIC: Low- and middle-income country
MOH: Ministry of Health
MOPH: Ministry of Public Health
NHS: National Health Service
OOP: Out-of-pocket
PGx: Pharmacogenomics
PM: Personalized medicine
PRISM: SingHealth Duke-National University Singapore Institute of Precision Medicine
SCAR: Severe cutaneous adverse reaction
SEA: Southeast Asia
SEAPharm: South East Asian Pharmacogenomics Research Network
TCELS: Thailand Center of Excellence for Life Sciences
THB: Thai Baht
UHC: Universal health coverage
UK: United Kingdom
US: United States
WHO: World Health Organization
