# Supplementary material for: Current landscape of personalized medicine adoption and implementation in Southeast Asia
Source: BMC Med Genomics. 2018 Oct 26;11:94. doi: 10.1186/s12920-018-0420-4 (PMC6203971; doi:10.1186/s12920-018-0420-4)
Supplement: Supplementary file 1 — Search strategy used in the scoping review. (DOCX 15 kb) [file 12920_2018_420_MOESM1_ESM.docx]

**Supplementary File 1.** Search strategy used in the scoping review

Searches were performed using broad combined search string in title and abstract fields as below: (pharmacogenetics OR pharmacogenomics OR genotyping OR “genetic screening” OR “genetic testing” OR genetic OR genomic OR “personalized medicine” OR “personalised medicine” OR “precision medicine” OR “genomic medicine” OR “stratified medicine” OR “individualized medicine” OR “individualised medicine”).
