# Supplementary material for: Current landscape of personalized medicine adoption and implementation in Southeast Asia
Source: BMC Med Genomics. 2018 Oct 26;11:94. doi: 10.1186/s12920-018-0420-4 (PMC6203971; doi:10.1186/s12920-018-0420-4)
Supplement: Supplementary file 2 — Six key themes in the assessment framework. (DOCX 18 kb) [file 12920_2018_420_MOESM2_ESM.docx]

**Supplementary File 2.** Six key themes in the assessment framework

| **Key themes** | **Indicators** |
| --- | --- |
| 1. Healthcare system | General:   - GDP per capita - Healthcare financing system - Healthcare expenditure per capita (USD) - THE (% of GDP) - Health coverage (%)   PM-specific:   - Presence of PM-related healthcare service delivery - Presence of PM-related healthcare workforce - Presence of PM-related healthcare information for patient, healthcare providers - Current availability of PM (genetic testing and/or treatment) - Financing mechanism for research, capacity-building, infrastructure, PM healthcare service - Monitoring review and evaluation of clinical, ethical, social, equity outcomes |
| 2. Governance | Definition  National strategy/plan including policies, initiatives  Comprehensive PM legislation/guideline  Ethical, social, legal (regulatory) framework on PM: research, marketing authorization, clinical use  Ethical, social, legal (regulatory) framework for genetic data: data ownership, privacy, security/protection, sharing  Direct to consumer test legislation or code of conduct  Multi-stakeholder working group/collaboration/partnership |
| 3. Access | HTA framework for coverage, pricing and reimbursement of PM (cost effectiveness, clinical effectiveness including analytical validity/clinical validity/clinical utility, ethical & social consideration) |
| 4. Awareness | Patient support/advocacy groups  Public awareness/health literacy  Patient involvement in healthcare and/or research |
| 5. Implementation | Integration to care pathway  Centre of expertise/excellence  Education and training for PM specialized and non-PM specialized healthcare workforce (primary healthcare) including medical school |
| 6. Data | ICT infrastructure, e.g. EHR  Biobank and patient registries  Data harmonization |
| **Abbreviations**  EHR, electronic health record; GDP, gross domestic product; HTA, health technology assessment; ICT, information and communication technology; PM, personalised medicine; THE, total health expenditure; USD, United States dollar. | |
