# Supplementary material for: Current landscape of personalized medicine adoption and implementation in Southeast Asia
Source: BMC Med Genomics. 2018 Oct 26;11:94. doi: 10.1186/s12920-018-0420-4 (PMC6203971; doi:10.1186/s12920-018-0420-4)
Supplement: Supplementary file 3 — List of articles/government documents/websites included in the scoping review. (DOCX 23 kb) [file 12920_2018_420_MOESM3_ESM.docx]

**Supplementary File 3.** List of articles/government documents/websites included in the scoping review

1. Electronic databases

1. Zayts O, Sarangi S, Thong MK, Chung BH, Lo IF, Kan AS, Lee JM, Padilla CD, Cutiongco-de la Paz EM, Faradz SM et al: Genetic counseling/consultation in South-East Asia: a report from the workshop at the 10th Asia pacific conference on human genetics. Journal of genetic counseling 2013, 22(6):917-924.

2. Padilla CD, Therrell BL: Newborn screening in the Asia Pacific region. Journal of Inherited Metabolic Disease 2007, 30(4):490-506.

3. Sukasem C, Chantratita W: A success story in pharmacogenomics: genetic ID card for SJS/TEN. Pharmacogenomics 2016, 17(5):455-458.

4. Bushyakanist A, Puangpetch A, Sukasem C, Kiertiburanakul S: The use of pharmacogenetics in clinical practice for the treatment of individuals with HIV infection in Thailand. Pharmacogenomics and Personalized Medicine 2015, 8:163-170.

5. Yamsri S, Sanchaisuriya K, Fucharoen G, Sae-ung N, Ratanasiri T, Fucharoen S: Prevention of severe thalassemia in northeast Thailand: 16 Years of experience at a single university center. Prenatal Diagnosis 2010, 30(6):540-546.

6. Chuansumrit A, Sirachainan N, Angchaisuksiri P, Sasanakul W, Wongwerawattanakoon P, Kadegasem P, Promsonthi P, Lamthean H: Establishment of carrier detection and prenatal diagnosis service for hemophilia in a developing country: Experience from international hemophilia training center-Bangkok. Blood 2012, 120(21):no pagination.

7. Piyamongkol W, Vutyavanich T, Piyamongkol S, Wells D, Kunaviktikul C, Tongsong T, Chaovisitsaree S, Saetung R, Sanguansermsri T: A successful strategy for Preimplantation Genetic Diagnosis of beta-thalassemia and simultaneous detection of Down's syndrome using multiplex fluorescent PCR. Journal of the Medical Association of Thailand 2006, 89(7):918-927.

8. Seguin B, Hardy BJ, Singer PA, Daar AS: Universal health care, genomic medicine and Thailand: Investing in today and tomorrow. Nature Reviews Genetics 2008, 9(SUPPL.1):S14-S19.

9. Schwarzer R, Rochau U, Saverno K, Jahn B, Bornschein B, Muehlberger N, Flatscher-Thoeni M, Schnell-Inderst P, Sroczynski G, Lackner M et al: Systematic overview of cost-effectiveness thresholds in ten countries across four continents. Journal of Comparative Effectiveness Research 2015, 4(5):485-504.

10. Ruangrit U, Srikummool M, Assawamakin A, Ngamphiw C, Chuechote S, Thaiprasarnsup V, Agavatpanitch G, Pasomsab E, Yenchitsomanus PT, Mahasirimongkol S et al: Thailand mutation and variation database (ThaiMUT). Human mutation 2008, 29(8):E68-75.

11. Yoshizawa G, Ho CWL, Zhu W, Hu CL, Syukriani Y, Lee I, Kim H, Tsai DFC, Minari J, Kato K: ELSI practices in genomic research in East Asia: implications for research collaboration and public participation. Genome Medicine 2014, 6.

12. Ngim CF, Lai NM, Ibrahim H: Counseling for prenatal diagnosis and termination of pregnancy due to thalassemia major: A survey of health care workers' practices in Malaysia. Prenatal Diagnosis 2013, 33(13):1226-1232.

13. Taib NA, Woo YL, Yoon SY, Kartini R, Thong MK, Yip CH, Teo SH: Early experience in a breast and ovarian cancer risk management clinic in Malaysia. Hereditary Cancer in Clinical Practice 2012, 10:no pagination.

14. Sasongko TH, Zabidi-Hussin Z, Othman NH, van Rostenberghe H: Informed consent template and guidelines on the ethical practice in human genetics and human genomic research; Initiatives of the universiti sains Malaysia. Malaysian Journal of Medical Sciences 2015, 22(6):47-53.

15. Zawawi M, Azmi I: Genetic Screening of Newborns in Malaysia: An Ethico-legal Prognosis. International Journal of Law Policy and the Family 2014, 28(2):135-149.

16. Azmi IM: Bioinformatics and genetic privacy: The impact of the Personal Data Protection Act 2010. Computer Law & Security Review 2011, 27(4):394-401.

17. Tan IK, Gajra B, Lim MS: Study of inherited metabolic disorders in Singapore - 13 years experience. Ann Acad Med Singapore 2006, 35(11):804-813.

18. Chieng WS, Lee SC: Establishing a cancer genetics programme in Asia - The Singapore experience. Hereditary Cancer in Clinical Practice 2006, 4(3):126-135.

19. Li ST, Yuen J, Zhou K, Ishak NDB, Chen Y, Met-Domestici M, Chan SH, Tan YP, Allen JC, Lim ST et al: Impact of subsidies on cancer genetic testing uptake in Singapore. Journal of Medical Genetics 2016:no pagination.

20. Sung C, Lee PL, Tan LL, Toh DS: Pharmacogenetic risk for adverse reactions to irinotecan in the major ethnic populations of Singapore: regulatory evaluation by the health sciences authority. Drug Saf 2011, 34(12):1167-1175.

21. Toh DS, Tan LL, Aw DC, Pang SM, Lim SH, Thirumoorthy T, Lee HY, Tay YK, Tan SK, Vasudevan A et al: Building pharmacogenetics into a pharmacovigilance program in Singapore: using serious skin rash as a pilot study. Pharmacogenomics J 2014, 14(4):316-321.

22. Wong ML, Chia KS, Yam WM, Teodoro GR, Lau KW: Willingness to donate blood samples for genetic research: A survey from a community in Singapore. Clinical Genetics 2004, 65(1):45-51.

23. Liu E: Cancer genomics: A revolution in cancer care. Asia-Pacific Journal of Clinical Oncology 2010, 6:138.

24. Zhou SF: Teaching of clinical pharmacogenetics for pharmacy students at the National University of Singapore. Pharmacy Education 2005, 5(3-4):235-240.

25. Chalmers D, Nicol D, Kaye J, Bell J, Campbell AV, Ho CW, Kato K, Minari J, Ho CH, Mitchell C et al: Has the biobank bubble burst? Withstanding the challenges for sustainable biobanking in the digital era. BMC medical ethics 2016, 17(1):39.

26. Chen J, Teo YY, Toh DS, Sung C: Interethnic comparisons of important pharmacology genes using SNP databases: potential application to drug regulatory assessments. Pharmacogenomics 2010, 11(8):1077-1094.

27. Tan EC, Loh M, Chuon D, Lim YP: Singapore human mutation/polymorphism database: A country-specific database for mutations and polymorphisms in inherited disorders and candidate gene association studies. Human Mutation 2006, 27(3):232-235.

28. Wong LP, Poh WT, Chan SL, Tan LL, Toh DS, Chia KS, Sung C, Teo YY: Singapore Pharmacogenomics Portal: a web resource for evaluating human genetic variations of genes responsible for drug responses. Pharmacogenet Genomics 2013, 23(6):329-332.

29. Chew MH, Tan WS, Liu Y, Cheah PY, Loi CT, Tang CL: Genomics of Hereditary Colorectal Cancer: Lessons Learnt from 25 Years of the Singapore Polyposis Registry. Annals of the Academy of Medicine, Singapore 2015, 44(8):290-296.

30. Manolio TA, Abramowicz M, Al-Mulla F, Anderson W, Balling R, Berger AC, Bleyl S, Chakravarti A, Chantratita W, Chisholm RL et al: Global implementation of genomic medicine: We are not alone. Science Translational Medicine 2015, 7(290).

1. Other sources
2. Chantratita W, Chantarangsu S, Kiertiburanakul S, Sungkanuparph S, Charoenyingwattana A: Integrating HIV-1 Pharmacogenomics into the Universal Coverage Health-Care System in Thailand: From Scientifc Evidence to Policy. J Pharmacogenom Pharmacoproteomics 2011, S6:001.
3. Shotelersuk V, Limwongse C, Mahasirimongkol S: Genetics and genomics in Thailand:

challenges and opportunities. Molecular Genetics & Genomic Medicine 2014, 210-216.

1. Ariani Y, Soeharso P, Sjarif DR: Genetics and genomic medicine in Indonesia. Molecular Genetics & Genomic Medicine 2017, 103-109.
2. Lee JMH, Thong MK: Genetic Counseling Services and Development of Training Programs in Malaysia 2013.
3. Md Yunus Z, Abdul Rahman S, Choy YS, Keng WT, Ngu LH: Pilot study of newborn screening of inborn error of metabolism using tandem mass spectrometry in Malaysia: outcome and challenges. J Pediatr Endocrinol Metab 2016, 29(9): 1031-1039.
4. Nik Hassan NN, Plazzer JP, Smith TD, Halim-Fikri H, Macrae F, A. Zubaidi AL et al: Harmonizing the interpretation of genetic variants across the world: the Malaysian experience. BMC Res Notes 2016, 9:125.
5. Mohd Saifuddeen S and Mohamad MH. Addressing genetic discrimination in the medical takaful system in Malaysia. Online J Res Islamic Studies 2014, 1(2).
6. Leong YH, Gan CY, Tan MAF, Abdul Majid MI. Present status and future concerns of expanded newborn screening in Malaysia: sustainability, challenges and perspectives. Malays J Med Sci 2014, 21(2):63-67.
7. Kementerian Kesehatan Republik Indonesia: Pedoman skrining hipotiroid kongenital SHK. In. Edited by RI KK; 2014.
8. The Indonesian National Genetic Database [<http://ethnos.findbase.org/home-id> ]
9. Ministry of Health Malaysia: National Thalassemia Screening Program. In. Edited by MOH. Putrajaya; 2008.
10. Malaysian Medical Council: MMC Guideline 010/2006 Medical genetics and genetic services. In.; 2006.
11. The Malaysian Cohort Biobank and the UKMMC-UMBI Biobank [http://www.ukm.my/umbi/biobank-sample-log-in/]
12. The Bioethics Advisory Committee Singapore: Genetic testing and genetic research. In.; 2005.
13. The National Medical Ethics Committee Singapore: Ethical guidelines for gene technology. In.; 2001.
14. Parliament Singapore: Guidelines for pre- and post-precision medicine counselling confidentiality. In.; 2017.
15. The GIS story [https://www.a-star.edu.sg/gis/About-Us/GIS-Story]
